# Trends in NHS doctor and dentist referrals to occupational health

**DOI:** 10.1093/occmed/kqw016

**Published:** 2016-02-28

**Authors:** D. Lalloo, E. Demou, E. B. Macdonald

**Affiliations:** ^1^Healthy Working Lives Group, Institute of Health and Wellbeing, College of Medical, Veterinary and Life Sciences, University of Glasgow, Glasgow G12 8RZ, UK,; ^2^MRC/CSO Social and Public Health Sciences Unit, Institute of Health and Wellbeing, College of Medical, Veterinary and Life Sciences, University of Glasgow, Glasgow G2 3QB, UK.

**Keywords:** Dentists, doctors, occupational health services, sickness absence.

## Abstract

**Background:**

Ill-health in doctors can affect performance and fitness to practice, and consequently patient care and safety, placing an important responsibility on National Health Service (NHS) occupational health (OH) services. Anecdotal discussions amongst NHS occupational physicians suggest an increase in the number of doctor attendances over time, with continuing focus on mental illness.

**Aims:**

To analyse OH referrals in doctors and dentists over 3 years.

**Methods:**

A retrospective evaluation of all doctor and dentist referrals to the OH service in one Scottish NHS board from April 2011 to March 2014, comparing this to management-reported sickness absence (SA) data held by the organization.

**Results:**

We found no significant change in overall OH referrals for doctors and dentists during the evaluation period. Mental illness was the commonest referral reason in all 3 years at 32, 38 and 30%, respectively, but no significant change in mental health referrals was demonstrated within the study period. SA events significantly increased during the three study years (356, 426 and 469, respectively; *P* < 0.05). OH referrals for those absent from work increased significantly between Years 1 and 3 (16 and 30, respectively; *P* < 0.05).

**Conclusions:**

SA events and OH referrals for those absent from work significantly increased between April 2011 and March 2014, but there was no commensurate (statistically significant) increase in overall OH referrals. These findings do not support anecdotal suggestions of increasing OH (or mental ill-health) attendances but can be used as a benchmark for other NHS organizations and for future trend comparisons.

## Introduction

The implications of ill-health in doctors are well recognized [1], particularly its effect on performance and fitness to practice, and consequently on patient care and safety. Doctors have difficulty admitting to illness [2,3] and health behaviours and barriers to doctors accessing appropriate healthcare have been the subject of much research [2,4,5]. Occupational health (OH) services play an important role in National Health Service (NHS) employee health and well-being, including doctors’ health [6]. Previous studies have shown that doctors seldom consult OH services [5,7], only doing so when forced by regulatory requirements. Distrust of OH services, particularly regarding confidentiality, has been highlighted [5,7]. Poor understanding of the expertise and support the specialty has to offer remains an issue, ironically possibly as a consequence of respecting the confidentiality of those attending. Throughput and use of OH services by doctors and dentists has previously been evaluated [8], highlighting that those studied did use OH services.

Anecdotal discussions amongst NHS occupational physicians within our service and at regional educational meetings suggested an increase in doctors’ attendance rates over time, with continuing focus on mental illness. We are not aware of any previously published studies evaluating trends in doctor and dentist referrals to OH services. The aim of this study was to analyse OH referrals in doctors and dentists over a 3-year period.

## Methods

We undertook a retrospective evaluation of doctor and dentist OH referrals from a Scottish NHS health board providing both acute and primary care services between April 2011 and March 2014. We evaluated both management referrals and self-referrals.

The OH service operates an electronic appointment system holding attendee information. Following each attendance, assessing OH clinicians record outcome data, including their opinion on work-relatedness and fitness for work. We extracted data for doctor and dentist referrals from this system and analysed them using SPSS Statistics 21. We anonymized the data at extraction and included only first management and self-referrals. Separately, we analysed management-reported sickness absence (SA) data held by the organization for the same period and compared them with the OH referral data. The health board’s research and development manager advised that formal ethics approval was not required because this was a service evaluation.

## Results

The study population was 951, 920 and 913 doctors and dentists for 2011, 2012 and 2013, respectively, out of ~12300 staff employed. Doctor and dentist referrals to the service for the 3 years are presented in Table 1. For the April–March cycle of 2011–2012, 2012–2013 and 2013–2014, respectively, 5% (50), 7% (60), and 7% (63), doctors and dentists were referred or self-referred to the service. These rates showed no significant difference over the 3 years. Of the 173 referrals, 93% (160) were doctors and 7% (13) were dentists, making the sample size of the latter insufficient to draw valid conclusions. Of the 160 doctor referrals, 54% (87) were female and over half (53%) were aged 40–59. General medical specialties (23%) were the commonest source of referrals, followed by surgery (18%) and psychiatry (14%). The 3-year summary data and data on referrals by disease and work-relatedness are presented in Table 2. No cases of multiple referrals were identified for any individual in each study year.

**Table 1.** Doctor and dentist (D&D) referrals to occupational health service (OHS) and management-reported absences

Absences and referrals to OHS					
	Total no. of D&D employed, *n*	Total referrals to OHS of D&D, *n* (%)	Management referrals D&D, *n* (D/D)	Self-referrals D&D, *n* (D/D)	No. of absences for D&D as reported by EASY [9], *n* (%)
2011–2012	951	50 (5)	31 (29/2)	19 (18/1)	356 (37)
2012–2013	920	60 (6)	43 (37/6)	17 (17/0)	426 (46)**
2013–2014	913	63 (7)	47 (41/7)	16 (16/0)	469 (51)**

EASY [9]—Early Access to Support for You.

**P < 0.01.

**Table 2.** Doctor and dentist referrals to OH service by disease category and work-relatedness (as considered by assessing OH clinician)

	Mental health, *n* (%)	Musculoskeletal, *n* (%)	Infection, *n* (%)	Skin, *n* (%)	Pregnancy, *n* (%)	Cardiac, *n* (%)	Surgical/ post-op, *n* (%)	Neurological, *n* (%)	Other, *n* (%)	Total, *n* (%)
April 2011–March 2012										
Caused by work	5 (31)	0 (0)	0 (0)	2 (50)	0 (0)	0 (0)	0 (0)	0 (0)	1 (20)	8 (18)
Made worse by work	7 (44)	0 (0)	0 (0)	1 (17)	4 (80)	0 (0)	0 (0)	0 (0)	0 (0)	12 (24)
Not work-related	4 (25)	10 (100)	3 (100)	2 (33)	1 (20)	0 (0)	4 (100)	2 (100)	4 (80)	30 (57)
April 2012–March 2013										
Caused by work	10 (43)	3 (21)	0 (0)	0 (0)	0 (0)	0 (0)	0 (0)	0 (0)	2 (18)	15 (25)
Made worse by work	7 (30)	4 (29)	0 (0)	2 (100)	0 (0)	0 (0)	0 (0)	0 (0)	1 (9)	14 (23)
Not work-related	6 (26)	7 (50)	3 (100)	0 (0)	3 (100)	2 (10)	1 (100)	1 (100)	8 (73)	31 (52)
April 2013–March 2014										
Caused by work	5 (26)	1 (11)	0 (0)	1 (25)	0 (0)	0 (0)	0 (0)	0 (0)	5 (28)	12 (19)
Made worse by work	8 (42)	3 (33)	0 (0)	1 (25)	0 (0)	1 (25)	0 (0)	1 (33)	2 (11)	16 (25)
Not work-related	6 (32)	5 (56)	6 (100)	2 (50)	0 (0)	3 (75)	0 (0)	2 (67)	11 (61)	35 (56)
Total: April 2011– March 2012	16 (28)	10 (26)	3 (25)	5 (50)	5 (63)	0 (0)	4 (80)	2 (33)	5 (15)	50 (28)
Total: April 2012– March 2013	23 (40)	14 (45)	3 (25)	2 (17)	3 (38)	2 (33)	1 (20)	1 (17)	11 (32)	60 (35)
Total: April 2013– March 2014	19 (33)	9 (29)	6 (50)	4 (33)	0 (0)	4 (67)	0 (0)	3 (50)	18 (53)	63 (37)
Total: over 3 years	58	33	13	11	8	6	5	6	33	173

Mental health (MH) conditions were the predom inant referral reason in all 3 years at 32, 38 and 30%, respectively. Musculoskeletal complaints were second followed by the ‘other’ category, which included work-related stress. As stress is not considered a medical condition [10], work-related stress referrals were recorded separately from MH conditions and comprised one, two and four for April–March 2011–2012, 2012–2013 and 2013–2014, respectively. We found no significant change in MH referrals over the study period, but SA events from the management-reported data (Table 1) significantly increased (356, 426 and 469, respectively; *P* < 0.01).

From the OH data, 43% (75) of the doctors and dentists were absent and 57% (98) were at work at the time of attendance. For those absent from work, there was a statistically significant increase in OH referrals between the first and third study years (16 and 30, respectively; *P* < 0.05). Year 2 referrals also increased (29) but did not reach statistical significance. There were no cases where the doctor was at work and the OH physician deemed them unfit to be so.

## Discussion

We identified a statistically significant increase in SA events and OH referrals for doctors and dentists on sick leave between April 2011 and March 2014 in one Scottish NHS board, but no statistically significant commensurate increase in overall OH referrals. Mental illness was the commonest reason for referral, but we found no significant difference in MH referrals over the 3 years.

A strength of this evaluation is its simplicity and that it involved assessment of descriptors such as absence status, work-relatedness and fitness for work. Limitations include the small sample sizes and lack of information on absence duration. The management-reported data did not allow extraction of the dentist data, so evaluation of doctors’ data alone was not possible.

The increase in OH referrals for those absent from work could reflect an increase in long-term absences, but we had no information about absence duration. The concordance between doctors’ own assessments of their fitness to be at work and the OH physicians’ assessments reflects positively in the mutual interest of doctor and patient care.

Our findings are unchanged from our previous evaluation and are consistent with other studies [2,8]. They do not support anecdotal suggestions of increasing OH, or mental ill-health, attendances. They can be used as a benchmark for other NHS organizations and for future trend comparisons. The discordance between OH attendances and SA events raises the question of whether doctors and dentists are using OH services as well as they might. Further work to evaluate this is needed, including investigating medical staff and managers’ adherence to SA policies, and absence duration profiles in this professional group.

**Key points**

- We identified a statistically significant increase in sickness absence events and occupational health referrals for doctors and dentists off sick during the 3-year study period, but no statistically significant commensurate increase in overall occupational health referrals.
- No significant change in mental health referrals was demonstrated over the 3 years.
- These findings do not support anecdotal suggestions of increasing occupational health, or mental ill-health attendances.

## Funding

Medical Research Council (partnership grant MC/PC/13027 to E.D.).

## Conflicts of interest

E.D. has previously received funding from Salus NHS Lanarkshire to conduct a service evaluation of Working Health Services Scotland (WHSS) (unrelated work).
